# Whole-Genome Sequencing Analysis of Listeria monocytogenes from Rural, Urban, and Farm Environments in Norway: Genetic Diversity, Persistence, and Relation to Clinical and Food Isolates

**DOI:** 10.1128/aem.02136-21

**Published:** 2022-03-22

**Authors:** Annette Fagerlund, Lene Idland, Even Heir, Trond Møretrø, Marina Aspholm, Toril Lindbäck, Solveig Langsrud

**Affiliations:** a Nofima, Norwegian Institute of Food, Fisheries and Aquaculture Research, Ås, Norway; b Department of Paraclinical Sciences, Faculty of Veterinary Medicine, Norwegian University of Life Sciences, Ås, Norway; The Pennsylvania State University

**Keywords:** *Listeria monocytogenes*, whole-genome sequencing, WGS, source tracking, persistence, molecular epidemiology, environmental pathogens, dairy farms

## Abstract

Listeria monocytogenes is a ubiquitous environmental bacterium associated with a wide variety of natural and human-made environments, such as soil, vegetation, livestock, food processing environments, and urban areas. It is also among the deadliest foodborne pathogens, and knowledge about its presence and diversity in potential sources is crucial to effectively track and control it in the food chain. Isolation of L. monocytogenes from various rural and urban environments showed higher prevalence in agricultural and urban developments than in forest or mountain areas, and that detection was positively associated with rainfall. Whole-genome sequencing (WGS) was performed for the collected isolates and for L. monocytogenes from Norwegian dairy farms and slugs (218 isolates in total). The data were compared to available data sets from clinical and food-associated sources in Norway collected within the last decade. Multiple examples of clusters of isolates with 0 to 8 whole-genome multilocus sequence typing (wgMLST) allelic differences were collected over time in the same location, demonstrating persistence of L. monocytogenes in natural, urban, and farm environments. Furthermore, several clusters with 6 to 20 wgMLST allelic differences containing isolates collected across different locations, times, and habitats were identified, including nine clusters harboring clinical isolates. The most ubiquitous clones found in soil and other natural and animal ecosystems (CC91, CC11, and CC37) were distinct from clones predominating among both clinical (CC7, CC121, and CC1) and food (CC9, CC121, CC7, and CC8) isolates. The analyses indicated that ST91 was more prevalent in Norway than other countries and revealed a high proportion of the hypovirulent ST121 among Norwegian clinical cases.

**IMPORTANCE** Listeria monocytogenes is a deadly foodborne pathogen that is widespread in the environment. For effective management, both public health authorities and food producers need reliable tools for source tracking, surveillance, and risk assessment. For this, whole-genome sequencing (WGS) is regarded as the present and future gold standard. In the current study, we use WGS to show that L. monocytogenes can persist for months and years in natural, urban, and dairy farm environments. Notably, clusters of almost identical isolates, with genetic distances within the thresholds often suggested for defining an outbreak cluster, can be collected from geographically and temporally unrelated sources. The work highlights the need for a greater knowledge of the genetic relationships between clinical isolates and isolates of L. monocytogenes from a wide range of environments, including natural, urban, agricultural, livestock, food production, and food processing environments, to correctly interpret and use results from WGS analyses.

## INTRODUCTION

Listeria monocytogenes is a bacterial pathogen responsible for the life-threatening disease listeriosis. The most common cause of listeriosis is considered to be ingestion of food contaminated by L. monocytogenes from unclean food production equipment (1, 2). L. monocytogenes is a ubiquitous environmental bacterium that has been associated with a wide variety of environments, such as rivers, soil, vegetation, wild and domesticated animals, food processing environments, and urban areas (3, 4). Consequently, a total absence of L. monocytogenes in non-heat-treated foods is difficult, perhaps impossible, to achieve. The literature is, however, not fully consistent about the main habitats of L. monocytogenes and the factors affecting its occurrence and spread to humans. It is therefore of importance to increase the understanding of the relationship between L. monocytogenes in natural and animal reservoirs, food processing environments, and human clinical disease.

The occurrence of L. monocytogenes in soil varies widely, from 0.7% to 45%, depending on the geographic area, season, and humidity (4–6). In comparative investigations, higher frequencies of L. monocytogenes have been found after rain, flooding, and irrigation events (7, 8). Several studies have reported high incidence of L. monocytogenes in water from rivers and lakes, with frequencies from 10 to 62% of the samples depending on the area and detection method (9–13). A link has been found between the proximity to upstream dairy farms and cropped land and the presence of L. monocytogenes in river water (10, 12). An explanation for this could be high frequencies of L. monocytogenes in feces from farm animals, e.g., cattle, ducks, and sheep, leaking into surrounding soil and water (9, 14, 15). Dairy farms are, for example, known to hold an L. monocytogenes reservoir, and prevalences in environmental samples of 11 to 24% have been reported (6, 15–17). However, L. monocytogenes is not particularly linked to farm animals and is frequently found in other animals and birds, such as game and urban birds, boars, garden slugs, and rodents (9, 18–21). An association between dense populations of humans and occurrence of L. monocytogenes in the environment has been reported. A U.S. study showed that 4.4% of samples from urban or residential areas contained L. monocytogenes, while the pathogen was less frequently found in samples from forests and mountains (1.3%) (22).

L. monocytogenes comprises four separate deep-branching lineages, which, from an evolutionary viewpoint, could be considered separate species (23). These are further subdivided by multilocus sequence typing (MLST) into sequence types (STs) and clonal complexes (CCs or clones). The lineage I clones CC1, CC2, CC4, and CC6 are reported to be associated with human disease, while lineage II clones CC9 and CC121 are strongly associated with food and food processing environments (24–28). While many studies have examined the molecular genotypes of L. monocytogenes isolates found in food, food processing environments, and clinical disease, much less is known about the diversity present in other environments. In the few published studies, the clonal diversity in environmental samples from soil and water appears to be very high, sometimes dominated by CCs associated with disease (CC1 and CC4), although other dominating clones (e.g., CC37) have also been observed (5, 13, 29). Several clones are also found in wild animals, e.g., CC7 and CC37, found in moose, boars, slugs, and game birds (18, 21, 30, 31). In environmental samples from dairy/cattle farms in Finland and Latvia, the lineage II clones CC11 (ST451), CC14, CC18, CC20, CC37, and CC91 were most predominant, while lineage I clones were rare (6, 32). Although there are some exceptions, e.g., CC1 being predominant in slugs collected in garden and farm environments in Norway (21), the majority of the clones identified in natural and farm environments do not seem to belong to CCs dominant among European food and clinical isolates.

Many studies have described persistence over time for L. monocytogenes clones in food processing facilities (2, 33, 34) and in individual cattle herds or farm environments (15, 35, 36). Whether L. monocytogenes can persist over long periods of time also in rural, urban, or agricultural environments has rarely been investigated. Studies of genetic relationships between L. monocytogenes isolates from natural and animal reservoirs and isolates from food and clinical sources are scarce. High-resolution molecular fingerprinting based on whole-genome sequencing (WGS) technology has revolutionized the ability to detect outbreaks and the presence of persistent strains (37). However, few studies have carried out WGS analyses of L. monocytogenes isolates collected from non-food-associated locations over the span of months and years. The present study aimed to use WGS to investigate the diversity and genetic relationships between L. monocytogenes isolates from rural, agricultural, and urban environments in Norway and to compare these with available data sets containing genomes of L. monocytogenes from human clinical and food-associated sources in Norway collected within the last decade.

## RESULTS

### Prevalence of L. monocytogenes in rural and urban environments in Norway.

**FIG 1:**
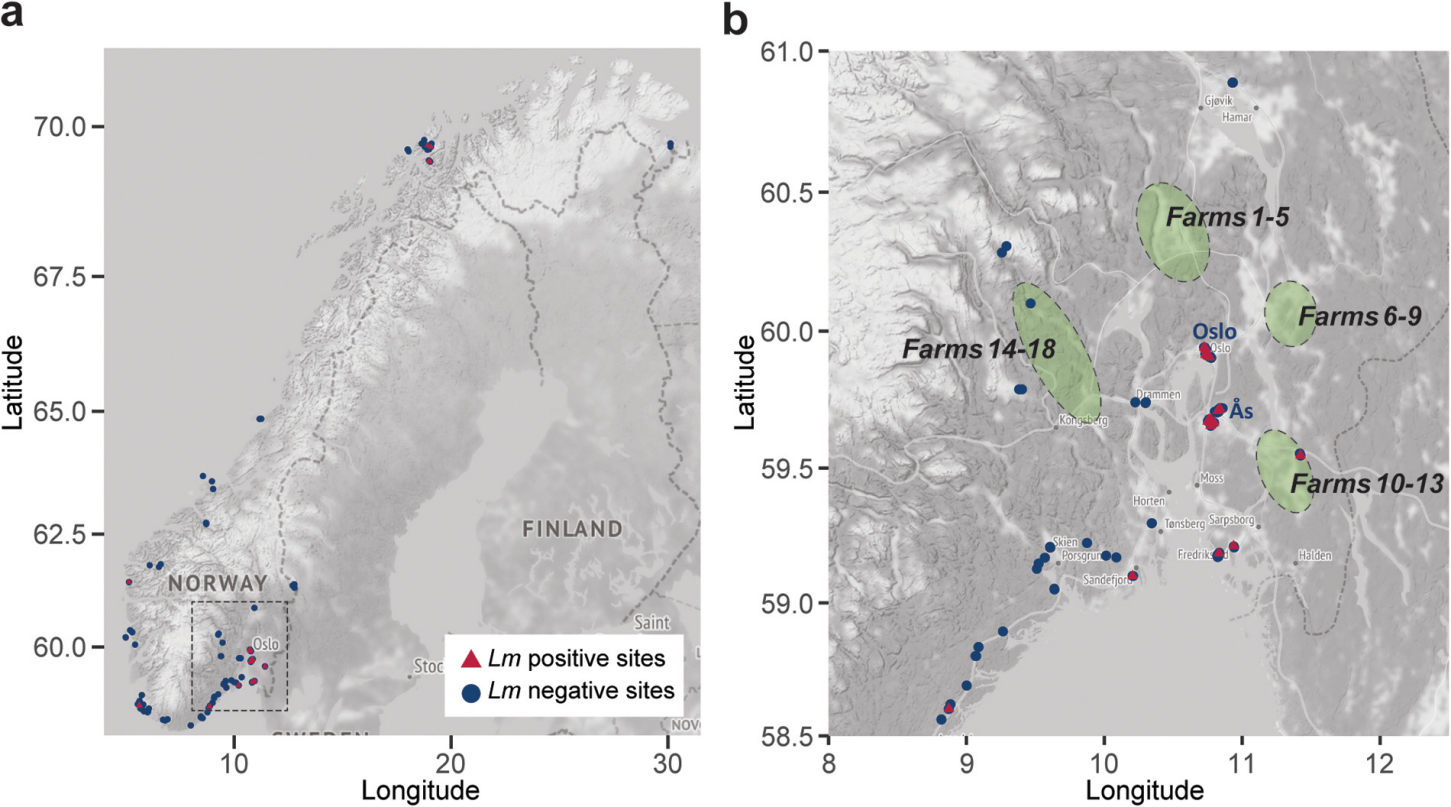
Maps showing the geographic location of sampling sites. (a) The location of sampling sites in rural and urban environments in Norway, with red triangles representing L. monocytogenes-positive samples and blue circles negative sampling points. The area outlined by the dashed square in panel a is the area shown in panel b. The green-shaded areas in panel b show the geographical origins of the dairy cattle farms sampled for L. monocytogenes in Idland et al. (16). The maps were plotted using the R package ggmap (66) using data from OpenStreetMap under the Open Database License (ODbL).

A total of 618 distinct environmental sites from rural and urban environments were sampled for L. monocytogenes between April 2016 and April 2020. The overall sampling scheme was designed to obtain an overview of the presence of L. monocytogenes in various habitats, and samples were collected from several geographical regions in Norway (Fig. 1a). To study potential persistence of L. monocytogenes clones over time, some sites were sampled more than once. At the onset of the study, we hypothesized that the presence of L. monocytogenes would be more strongly associated with farm animals, agricultural activity, and urban areas than with natural forests and other wildlands (22). During the first sampling occasion, 10% of sample sites were positive for L. monocytogenes (Table 1). In addition, 13 samples of commercial bags of plant soil or compost were negative for L. monocytogenes. In concordance with our hypothesis, the prevalence of L. monocytogenes was significantly higher in urban areas and in areas associated with agriculture and livestock (agricultural fields, grazelands, and animal paths) than in forest/mountain areas and on footpaths (*P < *0.02 by Fisher’s exact test). Sampling locations classified as footpaths were generally from nonurban areas, such as woods or other areas used for hiking. While 14% of urban areas were positive for L. monocytogenes, all samples from footpaths were negative for L. monocytogenes, and only 2% of the samples collected in woodland or mountain areas were positive.

**TABLE 1:** Prevalence of L. monocytogenes in rural and urban environments

Habitat or sampling area	No. of collected isolates	Prevalence of L. monocytogenes (%)
Grazeland or animal path	85	21
Urban or residential area	177	14
Agricultural field	70	11
Near food processing plant	106	10
Beach or sandbank	24	4
Forest or mountain area	121	2
Footpaths	35	0

### Detection of L. monocytogenes correlated with rain and sample humidity.

Previous studies have indicated that L. monocytogenes is more frequently isolated after recent rainfall, irrigation, and flooding events (7, 8). In the present study, 271 out of 618 samples were collected on days with rainfall and 347 samples on days with no rain within the previous 24 h. When collected on days with rain, 20% of samples were positive for L. monocytogenes, while on days with no rain within the last 24 h, only 3% of samples were positive. Thus, our data support previous studies suggesting that prevalence of L. monocytogenes is positively associated with rainfall (*P = *2 × 10^−12^ by Fisher’s exact test).

Upon sample collection, the humidity of the sampled material was categorized on a scale from 1 (completely dry) to 5 (liquid). Overall, the prevalence of L. monocytogenes in samples from the two driest sample categories was 5.6% (4/70) and 5.7% (11/196), while it was 17% (27/164) and 14% (12/86) in the more humid categories 3 and 4. The prevalence was significantly higher in the humid samples (categories 3 and 4) than in the two driest sample categories (*P < *0.02 by Fisher’s exact test). The prevalence in liquid samples (category 5) was 10% (10/102). Among the samples collected in urban environments, the sample humidity was not significantly associated (*P > *0.05) with the prevalence of L. monocytogenes, with an overall prevalence of 10% in categories 1 and 2 (10/92) and 17% in categories 3 to 5 (14/85).

### Persistent strains detected in rural and urban environments.

**FIG 2:**
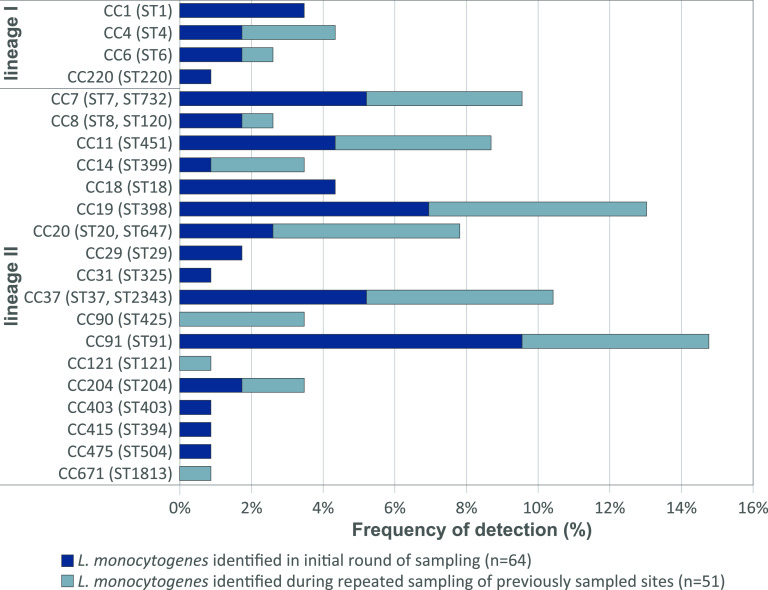
Distribution of CCs among identified isolates from rural and urban environments. The data are reported as percentages of the grand total number of isolates (*n *= 115). STs represented within each clonal complex (CC) are given in parentheses.

To examine whether environmental locations retained their status as L. monocytogenes positive or negative over time and whether the same clones were isolated repeatedly from the same location, 70 sites were subjected to one to three additional rounds of sampling the following years. In total, 115 L. monocytogenes isolates were collected in the current study (see Table S1 in the supplemental material). All isolates were subjected to WGS, *in silico* MLST, and whole-genome multilocus sequence type (wgMLST) analysis. The distribution of clones (CCs) among the identified isolates is presented in Fig. 2.

Of the 44 sampling points positive for L. monocytogenes in the first round of sampling, 28 sites (64%) were positive on at least one of the subsequent sampling occasions. Of the 26 initially negative sites, five turned out positive during later sampling events (19%), and one of these was positive twice. In total, 29 sampling sites were positive for L. monocytogenes more than once, and isolates belonging to the same ST were collected repeatedly from seven sites (Table 2). In six cases, STs repeatedly isolated from the same site were very closely related, with a maximum wgMLST allelic distance of 20. When also adjacent or slightly more distant sampling sites (maximum of 3 km) were included, a total of 14 clusters with genetic distances of <20 were repeatedly collected from the same location over periods ranging from 4 months to 3 years (Table S2 and Text S1). When the commonly employed core genome MLST (cgMLST) scheme described by Moura et al. (23) was employed, the isolates could not be distinguished, except in one cluster with distances of 0 to 1 cgMLST alleles. Twelve clusters, including two clusters each for CC91, CC11 (ST451), and CC37, represent clusters of highly similar isolates, with 0 to 8 wgMLST allelic differences. Together, the results strongly indicate that L. monocytogenes clones had persisted in the same environment or were repeatedly reintroduced between sampling events in both rural and urban locations.

**TABLE 2:** STs identified at sampling points positive for L. monocytogenes on repeated occasions^*a*^

Site no.	Sampling point description	2016, Oct	2017, Jun, Oct, Nov	2018, Jun	2018, Sept, Oct	2019, Sept	2020, Jan
Urban or residential area							
33	Brook in residential area	ST451				ST4	
48	Garden compost heap	ST451^a^				ST451^a^	ST425
49	Garden compost heap	ST451				ST425^b^	ST425^b^
66	Puddle next to road	ST20				ST4	
120	Flowerbed in town center		ST91		Negative	ST1813	ST398
121	In front of park bench by flowerbed		ST399		ST451	ST398^c^	ST39^c^
123	Grass lawn in town center		ST398^d^		ST398^d^	Negative	Negative
129	Roadside close to brook		ST37			ST91	Negative
251	Decaying leaves/vegetation on bike path		ST18			ST37^e^	ST37^e^
252	Soil near horse paddock		ST204			ST398	ST7
253	Along sidewalk curb		ST1			Negative	ST425
259	Flowerbed with pigeons, city center		ST204^f^			ST204^f^	ST398
262	At foot of tree, city center		ST6			ST204	ST120
268	Puddle on gravel path, city park		ST6			ST451	Negative
Grazeland or animal path							
53	Decaying vegetation by feeding station	ST4				ST399	ST37
54	Soil close to cattle feeding station	ST91				ST399	ST451
55	Mud close to cattle enclosure	ST37				ST399	ST91
56	Puddle of mud close to cattle enclosure	ST91				ST398	ST6
98	Sheep grazing pasture		ST398		ST451	ST2343	ST91
99	Sheep manure		ST398		Negative	Negative	ST91
100	Animal tracks by feeding station		ST398		Negative	ST37	Negative
101	Animal tracks by feeding station		ST398		Negative	ST91	Negative
130	Soil at edge of pond		ST91		Negative	ST121	Negative
133	Decaying vegetation at edge of pond		ST398		Negative	ST20^g^	Negative
134	Decaying vegetation at edge of pond		ST29		Negative	ST4	ST20^g^
Near food processing plant							
279	Grass next to cold storage entrance		ST732^h^	ST732^h^	ST732^h^		
287	Storm drain outside plant		ST1	Negative	ST647		Negative
363	Gravel from quay outside factory			Negative	ST732		ST647
406	Gravel from quay outside factory				ST1		ST647

a Allelic distance between isolates is indicated by the superscript letter: a, 3 wgMLST differences; b, 2 wgMLST differences; c, 2 wgMLST differences; d, 34 wgMLST differences and 0 cgMLST differences; e, 7 wgMLST differences; f, 3 wgMLST differences; g, 0 wgMLST differences (sites 133 and 134 are located 5 m apart); h, 0 to 4 wgMLST differences.

We also observed a case where a recent common contamination source was obscure: only 9 wgMLST alleles (and no cgMLST alleles) separated a pair of CC6 isolates found 30 km and 3 years apart; one isolate was from a grazing pasture in Akershus county in 2020, and the other was from soil by the root of a tree in Oslo city center in 2017.

### Persistence and cross-contamination on Norwegian dairy farms.

**FIG 3:**
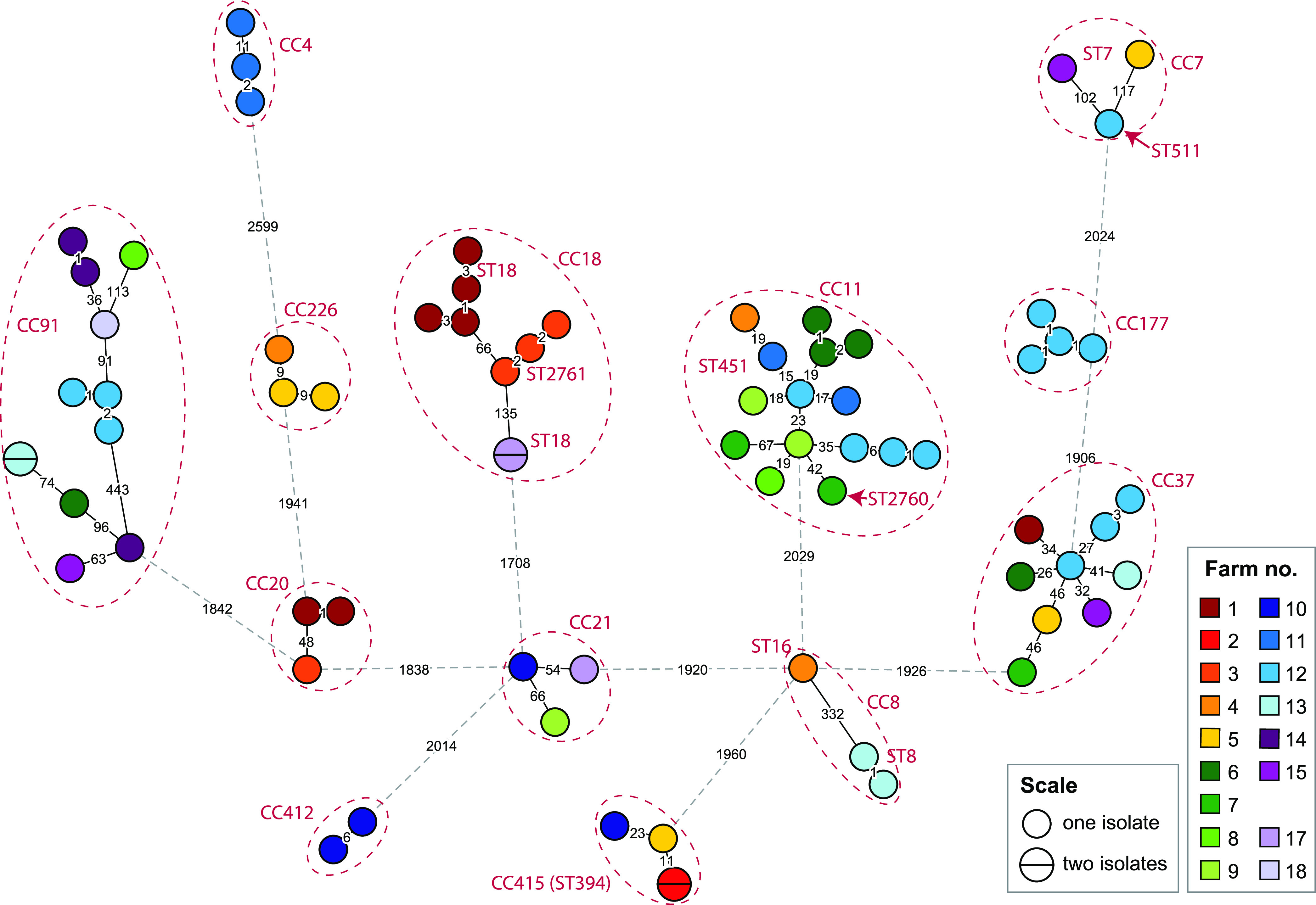
Phylogeny for the L. monocytogenes isolates from dairy farms. A minimum spanning tree based on wgMLST analysis is shown. The area of each circle is proportional to the number of isolates represented, and the number of allelic differences between isolates is indicated on the edges connecting two nodes. The CCs and STs are indicated next to each cluster (the CC number is the same as the ST number unless indicated otherwise). Edges shown as dashed lines separate clusters belonging to different clonal complexes. Isolates separated from the nearest other isolate by >1,700 wgMLST alleles (D011L, D058L, D080L, D084L, D144L, and D190L) were excluded from the figure for clarity.

In the next step, WGS was performed for a panel of 79 L. monocytogenes isolates collected from Norwegian dairy farms (16). A total of 18 dairy herds from four different geographical areas within a 100-km radius from downtown Oslo (Fig. 1b) had each been sampled four to six times between August 2019 and July 2020. Out of the 556 analyzed samples, L. monocytogenes was detected in 12 milk filters (13% prevalence), 30 cattle feces samples (30%), 32 samples of cattle feed (silage or silage mixture; 32%), and 5 teat swabs (5%). All bulk tank milk and teat milk samples were negative for L. monocytogenes, and for one of the farms (farm 16), all 34 collected samples were negative. An overview of the STs of the collected isolates (Table S1) is presented in Table 3, and a phylogenetic tree showing the genetic relationships between the individual isolates is shown in Fig. 3.

**TABLE 3:** L. monocytogenes STs identified on dairy farms^*a*^

Visit date	Sample	Farm no.																
		1	2	3	4	5	6	7	8	9	10	11	12	13	14	15	17	18
Aug/Sept 2019	MF			NS														
	Feces	NS				NS			ST91		ST11	ST4				ST7	ST21	
	Feed					ST226	ST37						ST37				ST6	
	TS															NS		
Nov/Dec 2019	MF	NS		NS	ST451			ST2760	NS			ST451	ST451					
	Feces	ST18			ST16	ST394		ST451			ST204			ST8				
	Feed	ST18			ST226	ST226	ST451	ST37					ST91					
	TS															ST37		
Jan 2020	MF	ST18							NS				ST37					
	Feces	ST18		ST2761									ST451				ST18	
	Feed	ST425	ST394	ST2761		ST7				ST224		ST4	ST451		ST91	ST91	ST18	
	TS			ST20														
Feb/Mar 2020	MF	NS						NS			NS	NS	ST177			NS		
	Feces	ST20		ST2761			ST451				ST21	ST451	ST177		ST91			
	Feed	ST37	ST394				ST451				ST412		ST177		ST91			
	TS	ST20																
May 2020	MF	NS	NS	NS									ST91	ST91			NS	
	Feces	NS	NS	NS									ST91	ST37			NS	
	Feed	NS	NS	NS									ST511	ST8			NS	ST91
	TS	NS	NS	NS									ST37	ST91			NS	
Jun 2020	MF	NS		NS			ST91		ST451	ST451								
	Feces	NS		NS		ST37				ST21	ST412	ST4	ST451	ST124			NS	
	Feed	NS		NS						ST451	ST394		ST177				NS	
	TS	NS	NS	NS					NS								NS	

a MF, milk filter; TS, teat swab; NS, not sampled; empty cells, negative for L. monocytogenes.

Twelve clusters, each comprising two to four isolates, with pairwise genetic distances in the range of 0 to 11 wgMLST alleles, were isolated from the same farm during repeated multiple visits over periods ranging from 2 to 10 months. These clusters involved 33 of the collected isolates and comprised 10 different CCs (Table S3). These observations strongly support previous studies indicating that the same L. monocytogenes clones can persist over time in individual cattle herds or farm environments (15, 35, 36).

Out of 12 isolates from milk filters, four belonged to a persistent cluster and one was closely related to an isolate from a teat swab sample obtained on the same sampling occasion. When the same clone was isolated from several sampling sites at the same farm, the pairwise genetic distances separating milk filter isolates from fecal, feed, or teat swab isolates ranged from 0 to 7 wgMLST allelic differences (Table S3). These links represent likely cross-contamination events where milk filters (and consequently milk) have been contaminated with L. monocytogenes clones found in the farm environment.

### Detection of closely related isolates from different geographic areas.

In four cases, closely related isolates belonging to CC11 (ST451), CC226, and CC415 (ST394) were collected from more than one dairy farm. The genetic differences between isolates from different farms were somewhat greater than the diversity between isolates found on the same farm, with between 9 and 20 pairwise wgMLST allelic differences. The number of cgMLST differences within each cluster was 0 or 1 (Table S3 and Text S1). These data indicate that farms located in different geographical areas host the same strain of L. monocytogenes.

Six clusters comprising L. monocytogenes from both dairy farms and isolates obtained from rural and urban environments were detected. The genetic distances separating isolates from the two data sets in these clusters ranged from 9 to 27 wgMLST allelic differences and 0 or 1 cgMLST differences (Table S4 and Text S1). The closest link was observed for a cluster of four CC37 isolates; two from grazing land/pasture in the vicinity of Ås and two from feed and teat swab samples obtained on two different visits to farm 12, located about 50 km east of Ås. The two pairs of isolates were separated by 9 to 14 wgMLST allelic differences and were indistinguishable by cgMLST.

To further explore the occurrence of genetic links between Norwegian isolates from natural and animal reservoirs, 24 of the 34 L. monocytogenes isolates collected from invading slugs (*Arion vulgaris*) from garden and farm environments in Norway by Gismervik et al. (21) were subjected to WGS analysis (Table S1). Interestingly, two pairs of slug isolates collected from different geographic locations showed only 2 (CC14) and 11 (CC1) wgMLST allelic differences. Furthermore, five clusters with 10 to 21 wgMLST allelic differences comprised a slug isolate and one or more isolates from either a rural/urban sampling site or from a dairy farm (Table S5). The closest genetic relationship concerned two CC1 isolates, in which a slug isolate from the west coast of Norway (collected in 2012) showed only 10 wgMLST allelic differences compared to an isolate collected from a street in a residential area in Oslo in 2017.

Thus, counting the previously mentioned pair of CC6 isolates collected in Akershus and Oslo, a total of 17 close genetic links between isolates collected at relatively distant geographic areas in Norway were detected in the set of 218 examined isolates. Presumably, not all clusters represent direct epidemiological links, especially in cases where isolates were collected several years apart. The observed genetic distances within the clusters, ≤21 wgMLST and ≤3 cgMLST allelic differences, are within the thresholds often suggested as an appropriate guide for defining an outbreak cluster, which is about 7 to 10 cgMLST differences (23, 38, 39) or about 20 single nucleotide polymorphisms (SNPs) in SNP analyses (40, 41), which have a sensitivity comparable to that of wgMLST (34, 42).

### Comparison with Norwegian clinical isolates.

The identification of close genetic links between isolates from different natural and animal-associated sources without known connections led us to hypothesize that it would be possible to identify clusters containing both environmental and clinical isolates with a similar level of genetic relatedness. A data set of Norwegian clinical isolates was identified, containing 130 genomes from 2010 to 2015 (92% of all reported cases in these years) (43) and two genomes from 2018 (ST20 and ST37), made publicly available by the European Centre for Disease Prevention and Control (ECDC) and the Norwegian Institute of Public Health (NIPH), respectively. Sequencing data of sufficient quality for wgMLST analysis was available for 111 of these isolates (Table S1). An initial comparison between the clinical isolates identified 15 pairs of isolates and nine larger clusters containing 3 to 12 isolates showing genetic distances of ≤10 cgMLST allelic differences (Table S6). Most clusters contained isolates collected during a time span of several years and could represent listeriosis outbreaks or epidemiologically linked cases.

**FIG 4:**
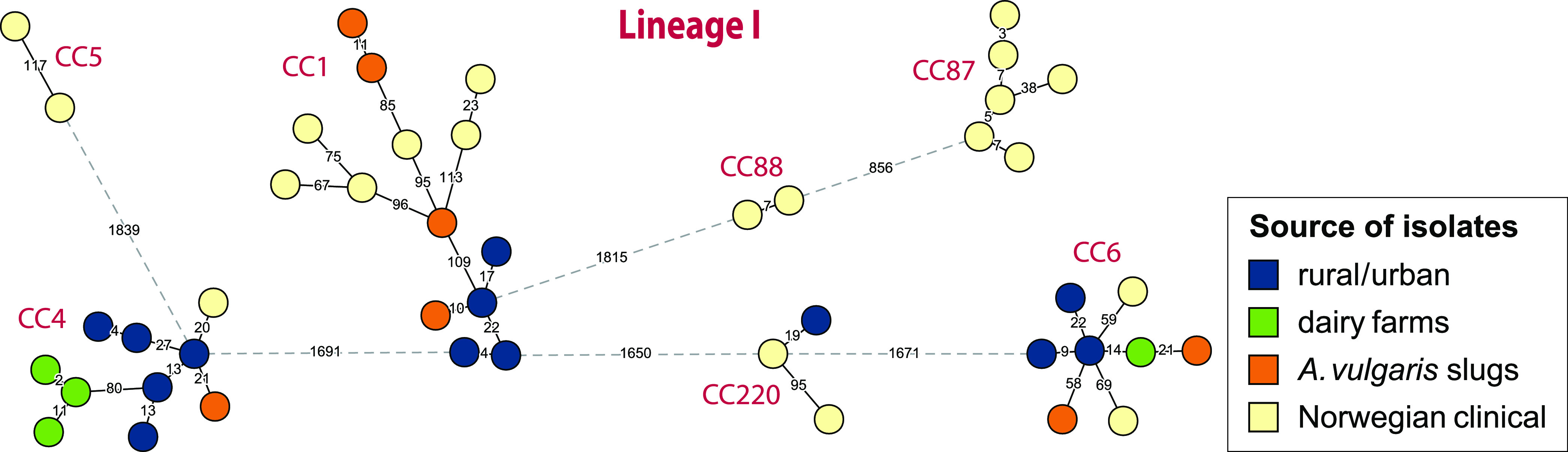
wgMLST phylogeny for L. monocytogenes lineage I isolates from Norway. Shown is a minimum spanning tree based on wgMLST analysis. The number of allelic differences between isolates is indicated on the edges connecting two nodes. Edges shown as dashed lines separate clusters belonging to different clonal complexes, which are indicated next to each cluster. Lineage I isolates separated from the nearest other isolate by >900 wgMLST alleles (D084L, ERR2522285, ERR2522267, and ERR2522291) were excluded from the figure for clarity.

**FIG 5:**
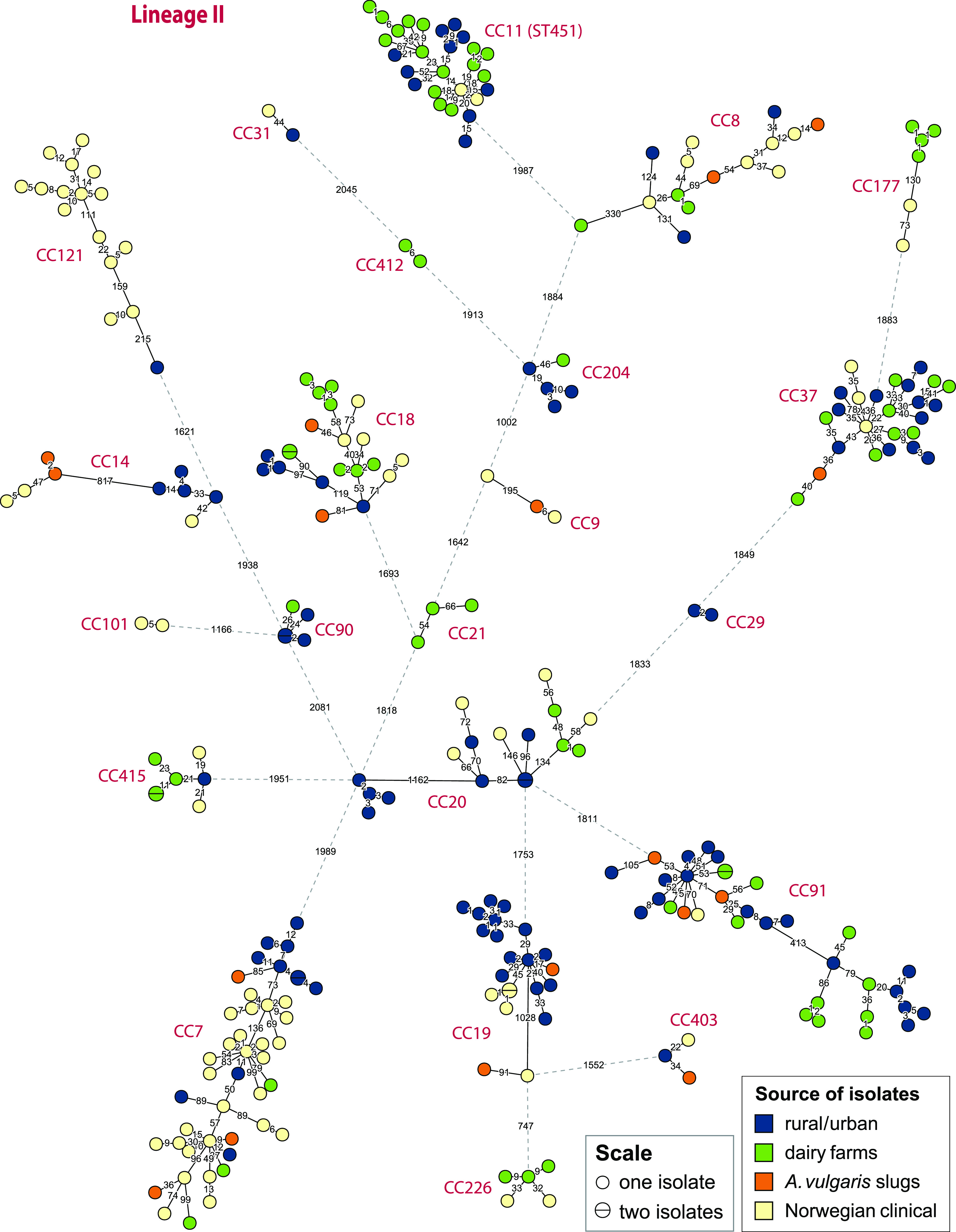
wgMLST phylogeny for L. monocytogenes lineage II isolates from Norway. Shown is a minimum spanning tree based on wgMLST analysis. The area of each circle is proportional to the number of isolates represented, and the number of allelic differences between isolates is indicated on the edges connecting two nodes. Edges shown as dashed lines separate clusters belonging to different clonal complexes, which are indicated next to each cluster. Lineage II isolates separated from the nearest other isolate by >900 wgMLST alleles (MF7614, MF6841, D144L, D190L, ERR2522251, and ERR2522298) were excluded from the figure for clarity.

A wgMLST analysis showing the genetic relationships between isolates originating from rural and urban environments, dairy farms, slugs, and clinical cases is shown in Fig. 4 and 5. Nine clusters contained clinical isolates differentiated from isolates sequenced in the current study by genetic distances in the range of 6 to 23 wgMLST allelic differences (0 to 7 cgMLST alleles) (Table S7 and Text S1). The environmental L. monocytogenes isolates closely related to clinical isolates were isolates from soil samples from both urban and rural locations (belonging to CC4, CC7, CC11/ST451, CC220, CC403, and CC415/ST394), three slug isolates obtained from garden and farm environments (CC7, CC8, and CC9), and a group of CC11/ST451 isolates from dairy farms. The closest genetic link was found between the single CC9 slug isolate (from 2012) and a clinical isolate from 2015, differentiated by only 6 wgMLST alleles (and 0 cgMLST alleles). The analysis shows that L. monocytogenes isolates that are genetically very closely related to clinical isolates can be detected in various natural and agricultural environments, even when isolates are collected across timespans ranging several years.

### Comparison of prevalence and diversity of MLST clones from different sources.

**FIG 6:**
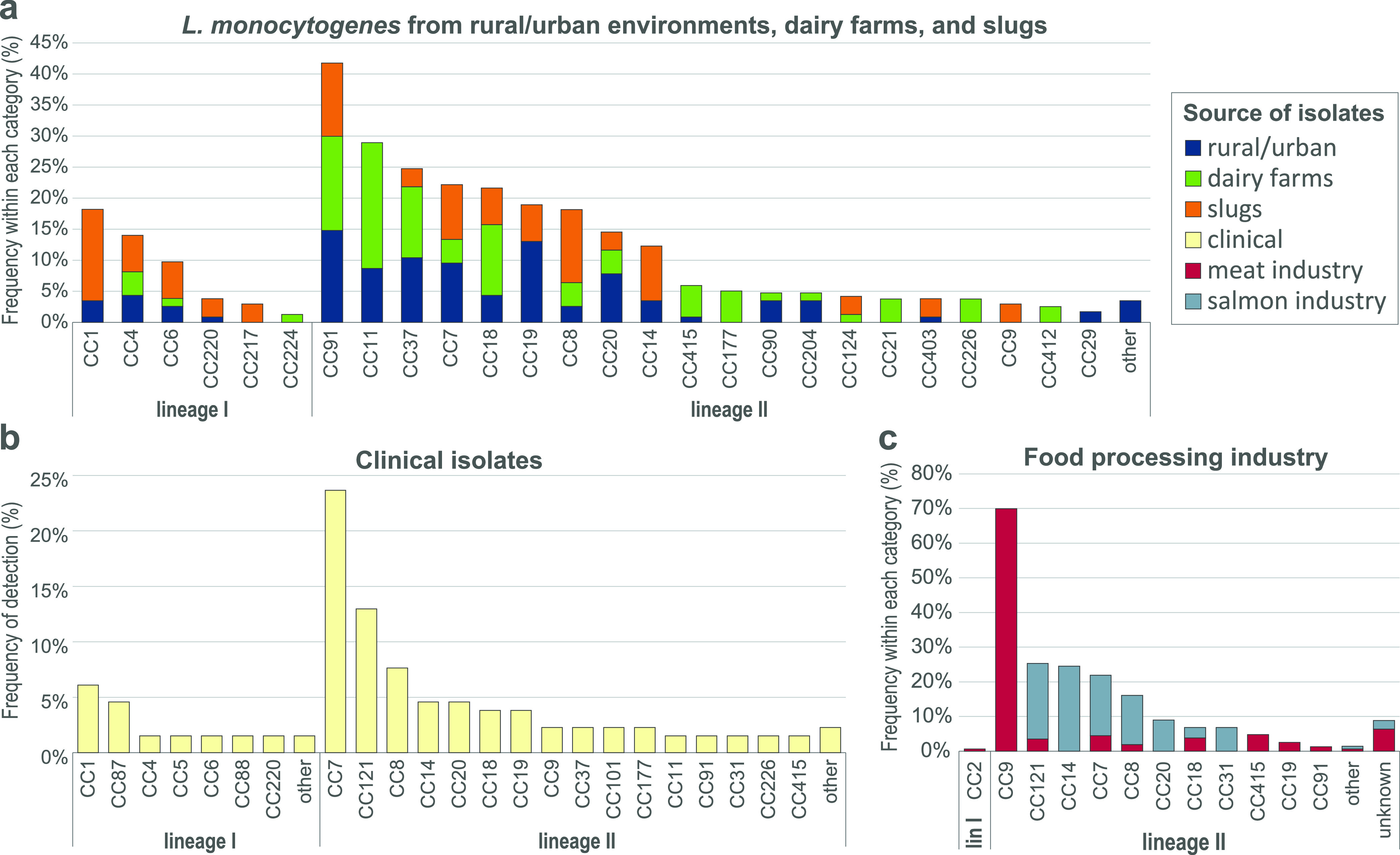
Prevalence and distribution of L. monocytogenes MLST clonal complexes (CCs) from different sample types in Norway. The data are reported as percentages of isolates within a given CC in each source category. (a) Prevalence in rural and urban environments (isolated during 2016 to 2020; *n *= 115 isolates), dairy farms (2019 to 2020; *n *= 87), and slugs (2012; *n *= 34). The “other” category comprises one isolate each for CC31, CC121, CC475, and CC671. (b) Prevalence in publicly available genomes from human cases of listeriosis in Norway (2010 to 2015; *n *= 129 and 2018; *n *= 2). The lineage I “other” category comprises a CC3 and a CC59 isolate, and the lineage II “other” category includes one isolate each for CC11, CC101, and CC177. (c) Prevalence within food processing facilities in Norway. The CCs were inferred for isolates from five meat and four salmon processing facilities (meat, 2012 to 2015, *n *= 293; salmon, 2011 to 2014, *n *= 358). The data used to predict the CC for each isolate were multiple-locus variable-number tandem repeat analysis (MLVA) obtained for all isolates and MLST data obtained for representative isolates from each obtained MLVA profile (45). The “unknown” category represents isolates with MLVA profiles identified only once and not subjected to MLST.

Most isolates from natural and agricultural environments belonged to L. monocytogenes lineage II, comprising 89%, 94%, and 68% of isolates from rural/urban environments, dairy farms, and slugs, respectively (Fig. 6a). The remaining isolates belonged to L. monocytogenes lineage I, as lineage III or IV isolates were not detected in the current study. The predominant clones among the rural/urban isolates were CC91 (15%), CC19/ST398 (13%), CC37 (10%), and CC11/ST451 (9%). No specific niches were found for these clones, as isolates were spread geographically (3 to 5 counties) and found in 3 to 5 different habitats/areas and in a range of humidity and weather conditions. CC91 appeared most ubiquitous, as it was isolated from five different counties, from different sample types (soil, sand, vegetation, and feces), from five different areas (agricultural fields, urban area, beach, grazeland, and forest), during all seasons, and from all categories of humidity. CC11/ST451, CC91, and CC37 were the most frequently isolated clonal groups at the dairy farms (18%, 15%, and 11%, respectively); each was detected on seven different farms. Among the slug isolates, the most common clones were CC1 (15%) and CC91 (12%) (21). A survey of previous studies indicated that CC1, CC7, and CC37 were the clones most commonly detected in various natural and farm environments (Table S8).

Among the examined Norwegian clinical isolates, CC7 was the most prevalent clonal group, accounting for 23% (*n *= 30) of the reported listeriosis cases, followed by CC121 (13%), CC8 (8%), and CC1 (6%) (Fig. 6b). In contrast to that observed in many other countries (27, 44), lineage I isolates composed a minority of the clinical isolates in this data set (20). The high prevalence of CC121 among the clinical isolates was unexpected, as this clone is commonly regarded as hypovirulent due to the frequent occurrence of premature stop codons (PMSC) in the gene encoding the virulence factor internalin A (*inlA*) (27), a characteristic also shared by the Norwegian CC121 clinical isolates. Interestingly, the single L. monocytogenes CC121 isolated in the current study, MF7617 from soil at the edge of a university campus pond in Ås, had an intact and presumably functional copy of *inlA*. This isolate was only distantly related to the clinical CC121 isolates, separated by 195 wgMLST alleles from the nearest clinical isolate. In contrast to CC121, the other three most commonly detected CCs among the Norwegian clinical isolates, CC1, CC7, and CC8, also were relatively common among the isolates from natural and agricultural environments, with each CC having an average prevalence of between 6% and 7.5% in the rural/urban, dairy farms, and slug isolate data sets (Fig. 6a).

Since listeriosis is primarily acquired from food, the frequency distribution of CCs for L. monocytogenes from the Norwegian food processing industry (Fig. 6c) was estimated from previous work encompassing 680 isolates from five meat and four salmon processing plants, collected during 2011 to 2015 (45). The prevalence of lineage I isolates was <1% among the food processing industry isolates, represented by only two CC2 isolates from the meat industry. In meat processing environments, CC9 was by far the most prevalent clonal group, representing 70% of isolates. It must, however, be noted that most of the collected isolates were from two intensively sampled processing plants (34). One slug isolate and three clinical isolates (2011, 2012, and 2015), but none from dairy farms or samples from rural and urban environments, belonged to CC9. In salmon processing environments, CC14 (ST14) was most prevalent (25%), followed by CC121 (22%), CC7 (ST7, ST732, and ST995; 18%), and CC8 (ST8 and ST551; 14%). CC14/ST14 was only represented by two clinical and two slug isolates and was not detected among isolates from rural/urban environments or dairy farms. The latter three were among the four most prevalent CCs among the Norwegian clinical isolates.

**FIG 7:**
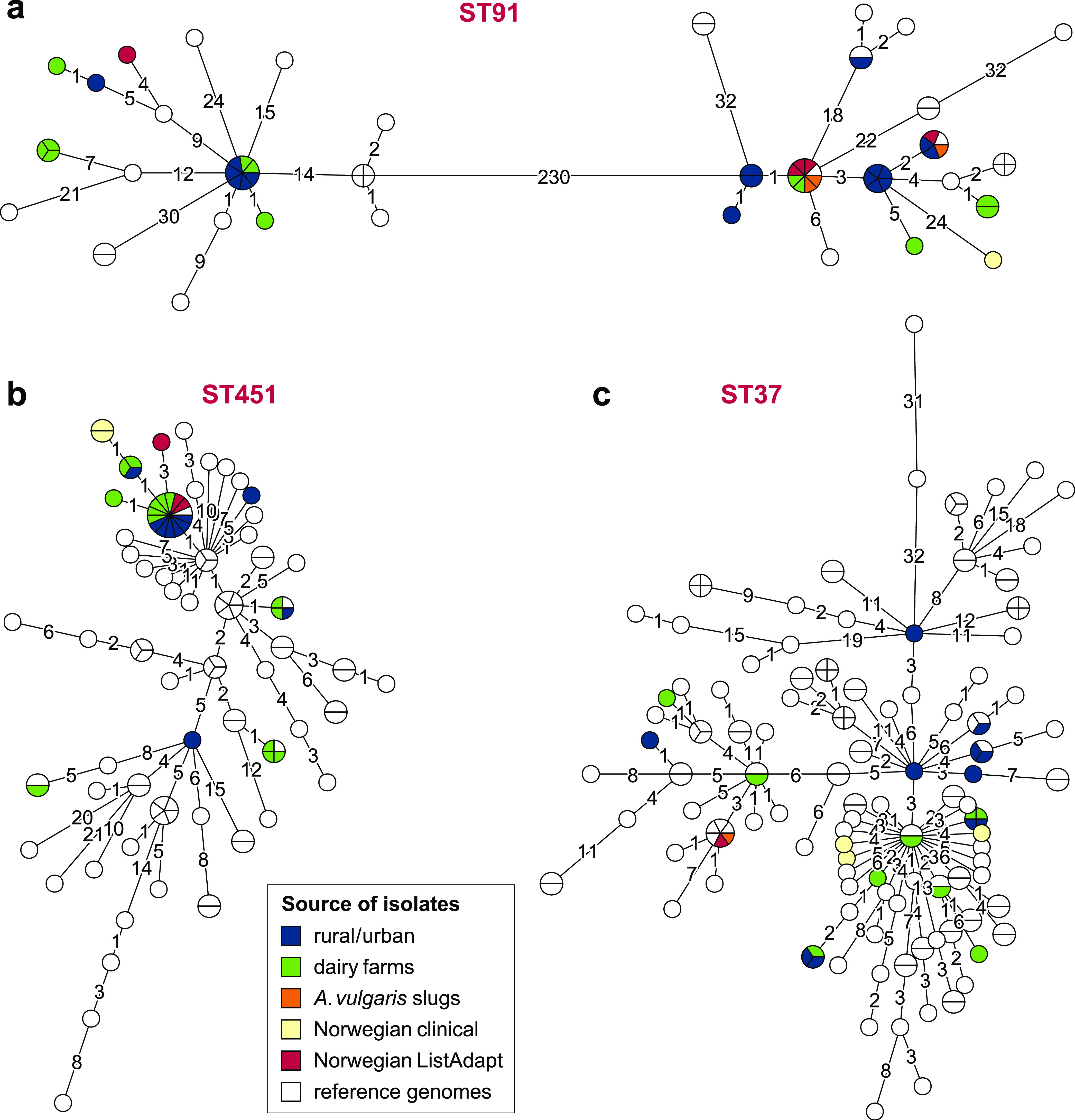
cgMLST phylogeny for the most common STs identified in the current study. Minimum spanning trees based on cgMLST allelic profiles for ST91 (a), ST451 (b), and ST37 (c), showing the relationship between the Norwegian isolates from natural environments, Norwegian clinical isolates, and reference genomes obtained from public databases. Reference genomes were obtained from the BIGSdb-*Lm* database hosted at the Pasteur Institute, WGS data from the EU project ListAdapt (also including genomes from Norwegian sources, labeled in red), and genome assemblies from NCBI GenBank. The area of each circle is proportional to the number of isolates represented, and the number of allelic differences between isolates is indicated on the edges connecting two nodes.

To examine the diversity of the most commonly detected L. monocytogenes clonal groups from Norwegian natural environments in an international context, a representative subset of reference genomes belonging to ST37, ST91, and ST451 were selected for comparative analysis using cgMLST (Fig. 7). Of the >6,000 examined publicly available genomes, 243 belonged to one of the three relevant STs. For ST91, a limited number of international reference sequences were available, and nearly 60% of the analyzed isolates were Norwegian. This suggests that this ST is more prevalent in Norway than in many other countries. For ST37, only limited clustering of Norwegian isolates relative to the international isolates was observed. In contrast, for ST91 and ST451, the Norwegian isolates appeared to cluster with isolates from other countries, indicating that they represent internationally dispersed clones.

## DISCUSSION

It has long been acknowledged that L. monocytogenes clones predominating among human clinical isolates differ from those that dominate in food (23, 24, 26, 44, 46, 47), and that persistent clones of L. monocytogenes may become established in food processing environments (2, 33, 34). Here, we show that the most ubiquitous clones found in soil and other natural and animal ecosystems (CC91, CC11, and CC37) are distinct from clones predominating among both clinical and food isolates, and that L. monocytogenes may persist and spread in urban and rural areas, grazeland, agricultural fields, and farm environments. The correspondence of major CCs was high for the three examined sets of environmental isolates (rural/urban, dairy farms, and slugs). CC37 appeared to be exceptionally widespread in natural environments and was isolated from nine different counties and a wide variety of habitats. It was also found to persist for years both at a farm and on a bike path in the capital of Norway. The ubiquity of this clone is also reflected by its detection in a large proportion of other studies investigating the identity of L. monocytogenes clones from natural and animal reservoirs, including wildlife, forest areas, and farms (5, 6, 13, 18, 21, 25, 29–31, 48).

The current study identified close genetic relationships between environmental isolates of L. monocytogenes collected from geographically and temporally unrelated sources despite a relatively low number of analyzed isolates. Although fixed clustering thresholds for defining outbreak clusters are controversial (37, 49, 50), the genetic distances in the observed clusters were well within the thresholds used to guide outbreak analyses (23, 38–41). In the majority of observed clusters, isolates with no known likely association were indistinguishable using cgMLST analysis, which is the method currently employed for surveillance of L. monocytogenes by many laboratories, including the Norwegian Institute of Public Health (51). This finding underscores the need for careful consideration of additional evidence, such as epidemiological data, traceback evidence, and phylogenetic tree topology, as part of WGS-based surveillance and outbreak investigations (52). Ideally, evaluation of possible epidemiological links should consider the occurrence of closely related strains in the whole food chain, including external contamination sources in urban and natural environments (50). Currently, a lack of published genomic data on L. monocytogenes from various sources is a barrier for effective management of this pathogen, both for public health authorities and for industrial actors.

During the last decade (2010 to 2020), an average of 24 yearly listeriosis cases have been reported in Norway, and most of them (80%) were domestically acquired (http://www.msis.no/). The implicated food is rarely identified. Only two outbreaks have been publicly reported during this period, both associated with traditional fermented fish (rakfisk), one in 2013 (ST802; four cases in Norway) (53) and one during the winter of 2018 to 2019 (ST20; 12 cases in Norway, 1 in Sweden [54]). A predominance of lineage II was observed among the Norwegian clinical isolates, comprising 80% of isolates during the years 2010 to 2015; an increase relative to the 56% observed during 1992 to 2005 (55). During 2010 to 2015, 71% of listeriosis patients were aged 70 or above, while during 1992 to 2005, only 42% of patients belonged to this high-risk age group (http://www.msis.no/). A distinct feature among Norwegian clinical isolates was the large proportion of CC121 isolates lacking functional internalin A. The only CC121 isolate collected from a natural environment did not have an *inlA* PMSC, supporting the hypothesis that *inlA* mutations constitute an adaptation to food industry environments (56). The relatively high proportion of cases caused by clones of a hypovirulent strain in Norway could be linked to national consumption and storage practices leading to sporadic ingestion of large numbers of the pathogen among high-risk groups.

Worldwide, the hypervirulent clones CC1 and CC4 are significantly more prevalent among clinical isolates than food isolates (27, 57, 58). Together, these two CCs constituted 8% of Norwegian clinical isolates and 11% of the isolates from natural and farm environments. CC1 and CC4 also appear to be prevalent in natural environments in other countries (13, 29). However, they were not detected in a study of L. monocytogenes in nine Norwegian food processing plants (45). Although at least 80% of meat, cheese, and fish consumed in Norway is produced domestically (59), imported processed foods remain a potential source of infections. Notably, however, 45% of Norwegian households report that they hunt, fish or collect bivalve molluscs, and about half of the population grow their own vegetables, herbs, or fruit and collect berries in the wild (60). Furthermore, the current study identified clusters containing closely related isolates from both clinical sources and natural environments despite comparing temporally nonoverlapping sets of isolates. Together, these observations suggest that the relative contribution of industrially processed foods to listeriosis infections is lower in Norway than in other countries.

## MATERIALS AND METHODS

### Sampling of L. monocytogenes from rural and urban environments.

Samples were taken to cover what was hypothesized as hot spots and cold spots for L. monocytogenes in the outer environment. The sampling plan was designed to cover different geographical regions of Norway and areas hypothesized to have high (urban areas, grazeland, animal paths, and areas near food processing factories) and low (forests and mountain areas, agricultural fields, beaches, and sandbanks) occurrence of L. monocytogenes. Samples classified as footpaths were generally from nonurban areas in woods or other areas used for hiking but were separately categorized, as we considered footpaths to be associated with human activities to a greater extent than more pristine woodland or mountain areas. A detailed sampling scheme was prepared, and convenience sampling was performed by people living in or travelling to different areas to cover Norway geographically and to get detailed results from specific areas (e.g., gardens) and local information. The sampling was performed by trained microbiologists informed about the objective of the study and which types of sites should be sampled. When possible, several different suspected hot and cold spots were sampled in the same geographical area, e.g., grazeland and a forest nearby where the cattle did not have access. Sampling was performed year-round except for winter. For a selection of sampling sites, sampling was repeated once or more over a period of 3 years.

The environmental samples (soil, sand, mud, decaying vegetation, surface water, animal dung, etc.) were collected in sterile 50-mL Nunc tubes. All sampling locations were photographed, and GPS coordinates, sample content, habitat/area, and weather conditions were recorded at the time of sample collection. Specific information about the sample was also noted, such as which animals the area was frequently exposed to (e.g., cattle, deer, sheep, and doves) and local information (e.g., popular areas for hiking). The humidity of the collected samples was assessed on a scale from 1 to 5, ranging from completely dry (1) to liquid (5). Samples were stored at 4°C for up to a week before processing, and analyzed according to ISO 11290-1 (61) with selective enrichment in half-Fraser and Fraser broth (Oxoid) and final plating on RAPID’*L.mono* agar (Bio-Rad).

### Whole-genome sequencing.

For each L. monocytogenes isolate from rural/urban environments or from *Arion vulgaris* slugs (21), a single colony was picked, inoculated in 5 mL brain heart infusion broth, and grown at 37°C overnight. Culture samples (1 mL) were lysed using lysing matrix B and a FastPrep instrument (both MP Biomedicals), and genomic DNA was isolated using the DNeasy blood and tissue kit (Qiagen). Libraries for genome sequencing were prepared using the Nextera XT DNA sample preparation kit (Illumina) and sequenced using 2 × 300 bp reads on a MiSeq instrument (Illumina).

Colonies from the L. monocytogenes isolates from dairy farms (16) were inoculated in 20 mL tryptone soy broth and incubated at 37°C for 24 h before 1 mL was pelleted and DNA extracted using the DNeasy blood and tissue kit (Qiagen). Libraries for genome sequencing were prepared using the NEBNext Ultra DNA library prep kit (New England Biolabs) with random fragmentation to 350 bp and sequencing of 2 × 150 bp on a NovaSeq 6000 S4 flow cell (Illumina).

### Genome assembly.

All genome assemblies used in phylogenetic analyses were generated as follows. Raw reads were filtered on q15 and trimmed of adaptors before *de novo* genome assembly was performed using SPAdes v3.10.0 or v3.13.0 (62), with the careful option and six *k*-mer sizes (21, 33, 55, 77, 99, and 127). Contigs with sizes of <500 bp and with coverage of <5 were filtered out. For the L. monocytogenes isolates from dairy farms, the genomes released to NCBI GenBank as accession no. PRJNA744724 (see “Data availability,” below) were generated using SPAdes v3.14.1 incorporated in the software tool Shovill, available at https://github.com/tseemann/shovill. Shovill also performed adaptor trimming using Trimmomatic, corrected assembly errors, and removed contigs with sizes of <500 bp and coverage of <2. The quality of all assemblies was evaluated using QUAST v5.0.2 (63) (see results in Table S9 in the supplemental material).

### Phylogenetic analyses.

Classical MLST analysis followed the MLST scheme described by Ragon et al. (64) and the database maintained at the Institute Pasteur's L. monocytogenes online MLST repository (https://bigsdb.pasteur.fr/listeria/). *In silico* MLST typing was performed for raw sequencing data using the program available at https://bitbucket.org/genomicepidemiology/mlst (65) and for genome assemblies using the program available at https://github.com/tseemann/mlst. CCs are defined as groups of ST profiles sharing at least six of seven genes with at least one other member of the group, except for CC14, which is divided into CC14, represented by ST14 and ST399 in the current work, and CC91, represented by ST91, as isolates belonging to these two groups do not cluster in phylogenetic analyses of L. monocytogenes populations (27).

The wgMLST analysis was performed using a whole-genome scheme containing 4,797 coding loci from the L. monocytogenes pangenome and the assembly-based BLAST approach, implemented in BioNumerics 7.6 (https://www.bionumerics.com/news/listeria-monocytogenes-whole-genome-sequence-typing). The cgMLST analysis was performed using the scheme described by Moura et al. (23), which is a subscheme of the wgMLST scheme employed in the BioNumerics platform. For publicly available genomes (described below), cgMLST profiles were obtained by sequence query against the BIGSdb-*Lm* cgMLST allele database maintained at the Institut Pasteur (https://bigsdb.pasteur.fr/listeria/). For the genomes sequenced in the current study, cgMLST profiles were extracted from the wgMLST profiles by mapping of the sequences of the cgMLST allele subset to the publicly available nomenclature through synchronization of BioNumerics with the BIGSdb-*Lm* cgMLST allele database. A subset of isolates was subjected to cgMLST analysis using both approaches to confirm that identical cgMLST profiles were obtained. During wgMLST analysis in BioNumerics, each identified unique allele sequence is designated an allele identifier integer. In contrast, for analyses involving the BIGSdb-*Lm* cgMLST allele database, only alleles that are already present in the database will be identified and receive an allele identifier, while novel alleles are recorded as missing loci.

Minimum spanning trees were constructed using BioNumerics based on the categorical differences in the allelic cgMLST or wgMLST profiles for each isolate. The number of allelic differences between isolates was read from genetic distance matrices computed from the absolute number of categorical differences between genomes. Loci with no allele calls were not considered in the pairwise comparison between two genomes. The criterion for inclusion of a cluster in Table S2, Table S3, Table S4, Table S5, and Table S7 was that each genome included in the cluster showed ≤20 or ≤21 wgMLST allelic differences toward at least one other genome in the cluster. For Table S6, clusters comprising isolates showing ≤10 cgMLST allelic differences toward at least one other genome in the cluster were included. Consequently, for clusters with three or more genomes, individual pairs of genomes with genetic distances exceeding the set thresholds were included in the clusters (see also Text S1).

### Publicly available genomes.

Available genomes of clinical isolates from human patients in Norway were identified by searching the NCBI Pathogen Detection database (https://www.ncbi.nlm.nih.gov/pathogens) on 30 August 2021. Available raw sequencing data from NCBI BioProjects submitted by ECDC and NPIH, accession numbers PRJEB26061 (43) and PRJEB25848, were subjected to *de novo* genome assembly as described for isolates from rural/urban environments. *In silico* MLST genotyping was successful for all genomes except one of the genomes published by the ECDC, and wgMLST analysis was successful for all except 21 of the ECDC genomes.

Reference genomes included in the cgMLST analysis of ST37, ST91, and ST451 genomes were identified from the following selected sources on 27 August 2021: (i) cgMLST profiles from the BIGSdb-*Lm* database (https://bigsdb.pasteur.fr/listeria/), with 15 genomes belonging to relevant STs; (ii) raw WGS data from the ListAdapt project (https://onehealthejp.eu/jrp-listadapt/), containing 1,552 genomes (BioProject no. PRJEB38828); *de novo* genome assembly was performed for the 165 genomes of relevant STs; and (iii) genome assemblies from NCBI GenBank; among the 3,926 L. monocytogenes genomes, 63 genomes belonged to the relevant STs.

### Data availability.

Data from this whole-genome shotgun project have been deposited in the NCBI GenBank database under BioProject numbers PRJNA689486, PRJNA744724, and PRJNA689487. For GenBank and Sequence Read Archive (SRA) accession numbers, see Table S1. The assemblies were annotated using the NCBI Prokaryotic Genomes Automatic Annotation Pipeline (PGAAP) server (http://www.ncbi.nlm.nih.gov/genome/annotation_prok/).
